# To Mix or Not To Mix? A Meta-Method Approach to Rethinking Evaluation Practices for Improved Effectiveness and Efficiency of Psychological Therapies Illustrated With the Application of Perceptual Control Theory

**DOI:** 10.3389/fpsyg.2019.01445

**Published:** 2019-06-26

**Authors:** Timothy A. Carey, Vyv Huddy, Robert Griffiths

**Affiliations:** ^1^ Centre for Remote Health, Flinders University, Alice Springs, NT, Australia; ^2^ Clinical Psychology Unit, University of Sheffield, Sheffield, United Kingdom; ^3^ Greater Manchester Mental Health NHS Foundation Trust, Manchester, United Kingdom; ^4^ University of Manchester, Manchester, United Kingdom

**Keywords:** randomized controlled trial, perceptual control theory, effectiveness, efficiency, internal validity, test for the controlled variable

## Abstract

Progress in the development of more effective and efficient psychological therapies could be accelerated with innovative and nuanced approaches to research methodology. Therapy development has been dominated by a mono-methodology attitude with randomized controlled trials (RCTs) regarded as a “gold standard” despite the concept of a single methodology being ascribed gold standard status having been called into question. Rather than one particular methodology being considered superior to all others, the gold standard approach should be matching appropriate methodologies to important research questions. The way in which that matching should occur, however, is far from clear. Moving from a mono-methodological approach to mixed-method designs has not been straightforward. The ways in which methods should be mixed, to arrive at robust and persuasive answers to genuine research questions, is not entirely clear. In this paper, we argue that attention to the meta-methods underpinning all research designs will improve research precision and provide greater clarity about the contribution of any particular program of research to scientific progress in that field. From a meta-method perspective, the matter of *what* changed can be delineated from *why* or *how* these changes occurred. Different methods and different types of mixing can be justified for each meta question. A meta-method approach should make explicit the assumptions that guide the development of research designs and also promote the articulation of putative mechanisms that might be relevant. By paying greater attention to assumptions such as how causality occurs, and important mechanisms of change, the mixing of methodologies that are still not mainstream in this area such as routine outcome monitoring and evaluation and functional model building, can occur. By adopting methodologies that focus on learning about a program’s strengths and weaknesses rather than presiding over judgments of whether or not the program is deemed to be effective, we will move much closer to a position of being able to understand what programs under which conditions people find most helpful for their purposes.

There is little doubt that the provision of psychological treatment is extremely beneficial for many people a lot of the time (Cuijpers et al., 2008). There is equally as little doubt, however, that both the effectiveness and efficiency of treatments could be improved. To a very large extent, the development and evaluation of psychological treatments has relied almost exclusively on a mono-methodological approach, with the Randomized Controlled Trial (RCT) being afforded a privileged position in comparison to alternative methodologies. Despite RCTs being regarded as a “gold standard” methodology and an exponential increase in the number of RCTs of psychological treatments being conducted in the decades since 1980, evidence indicates that both the effectiveness and efficiency of psychological treatments is decreasing rather than increasing (Carey et al., 2017; Weisz et al., 2018), though the interpretation of this finding has been taken to reflect improving methods (Ljótsson et al., 2017).

When scientific activity is deployed to solve a problem, it is generally not considered acceptable to apply rigorous and improved research methods and move *further* from a solution, rather than closer to it. It is difficult to understand the argument, therefore, that, even though our research methods are *improving*, the effectiveness of our treatments, and, by implication, the helpfulness to patients, is *decreasing.* Clearly, a different approach is required in terms of researching what the best solution might be, and how to rigorously and impartially test whether it is effective.

We argue, in this paper, that the different approach that is required is a difference in *kind* as well as in content. We are not arguing simply for switching from RCTs to some other methodology. Nor are we advocating that we should necessarily change from a mono-methodological approach to a mixed-method approach. While mixed-method designs have become increasingly popular and are capable of providing rich data, they suffer from the same problems as mono-methodological approaches when they are used indiscriminately. The quality of mixed-method research is not always easy to determine. Furthermore, there are currently few guidelines or protocols to assist researchers in how to most appropriately mix different methodologies. The United Kingdom Medical Research Council produced pragmatic guidance for those developing complex interventions (Medical Research Council, 2000). While this guidance offers questions that researchers can use to guide their research decision making, along with illustrative case studies, it takes an a-theoretical perspective on the practical steps required to evaluate interventions.

Our position in this paper is that, greater attention must be paid to the “meta-methods” underpinning all research designs. We use the term “meta-methods” to refer to a researcher’s implicit or explicit assumptions about the nature of reality (ontological assumptions) and how that reality can be known (epistemological assumptions), as well as how these assumptions subsequently inform decisions about the most appropriate methodology or methodologies (research strategy) to use and the specific strategies to be employed. It is conventional for researchers conducting qualitative methods to reflect the ways in which their values, experiences, and theoretical and ideological stances have shaped the research process (Berger, 2015). Because of the theoretical assumptions of quantitative research, which have their origins in positivism (Sale et al., 2002), the same level of attention has not been paid to these meta-method issues. For the reasons outlined below, we argue that meta-method considerations should explicitly inform the decision-making process about when and how to mix quantitative and qualitative methods.

We begin by illustrating problems with a mono-methodological approach by highlighting how a commonly held assumption, that RCTs allow causality to be attributed specifically to *interventions* (Sprott and Farewell, 1993), has impeded the development of increasingly effective and efficient psychological treatments. Throughout this discussion, we will highlight relevant meta-method considerations and then, in the second half of the paper, we will outline the meta-method approach in greater detail. In the first part of the paper, we focus on RCTs specifically only because they are an extremely common and an especially highly regarded methodology. It is the general principles we regard as important, however, rather than the way they are applied specifically to RCTs.

We will also propose in this paper that a theory of human behavior, Perceptual Control Theory (PCT), provides a useful basis for meta-method decision-making. When researchers pay more direct attention to the meta-method assumptions *that are already* guiding their decision-making processes with regard to important research decisions, research might become more creative but also more robust and compelling. Research will also become part of a more complete scientific process in which the results of the research are used to inform theories and assumptions (Piantadosi, 2005) about the ways in which a particular aspect of the world “works.” The ultimate consequence of creating a cyclical relationship between theory and the research process will be psychological treatments that enable people to create the outcomes they desire with greater effectiveness and efficiency.

It should be stated explicitly at this point that we are making an assumption in this paper that the researchers go about their business in the ways that they do in order to achieve certain purposes. These purposes are related to the beliefs, attitudes, and values that researchers hold with regard to the acquisition of knowledge and the nature of human functioning. These beliefs and attitudes might be implicit rather than explicit. They might also not be fully formed or reasoned. Nevertheless, it is our contention that they exist in some form and have an important role in determining the way in which research is conducted.

## Problems with Assigning Causation to Interventions or Treatments

Methodological problems with RCTs of psychological treatments are well recognized and longstanding (Jadad and Enkin, 2007; Carey and Stiles, 2016). A problem that is perhaps more important, but also less well recognized, concerns the statement about causation which underpins the RCT methodology. Causality is a meta-method factor that researchers should be required to *explicitly* address when designing programs of research. The causality mechanism underpinning RCTs, however, is rarely explicitly discussed, even though RCTs have been described as “Galilean experiments” (Cartwright, 2010, p. 65). The Galilean term is used to illustrate how the operation of the cause in the absence of interfering factors is analogous to how two objects in a gravity field attract each other with a single force that can be derived from Newtonian principles. As we later make clear, however, this meta-method assumption is questionable when considering that people in research studies are purposeful entities and not inanimate objects moving in space. Meta-method assumptions become more problematic when these assumptions are held implicitly and can lead to methodological decisions that are erroneous.

The fundamental purpose of an RCT is to establish that a particular product or agent *causes* certain results reliably and unambiguously (Sprott and Farewell, 1993). It is causation that the “R” and the “C” are primarily used to address. Random allocation (the “R”) and controlled conditions (the “C”) are two examples of the meta-method assumptions we are highlighting in this paper. Both of these methodological strategies are instructive in the context of meta-method assumptions because they each have nuances that influence the credibility of research results. Random allocation, for example, does not always occur according to the variables that are important in the delivery of psychological treatments (Carey and Stiles, 2016). This is not a comment on the way in which the allocation process is conducted. Rather, it is a statement about the *relevance* of the variables that guide this allocation. If the purpose of implementing an allocation process is to remove bias and ensure comparability between groups (Suresh, 2011), then it is important to ensure that the groups are comparable according to the variables that are likely to be important in the study. Even when allocation does occur according to relevant variables, however, it needs to be remembered that random allocation is only effective, in terms of establishing equivalence between groups, *on average* across many studies. Despite comparability being a stated purpose of random allocation, this process does not guarantee equivalence of groups *for any particular study*.

The strategy of controlled conditions to improve internal validity has introduced additional problems in terms of creating an implementation gap between experimental studies and routine clinical practice (Carey et al., 2017). Moreover, neither random allocation nor establishing controlled conditions addresses the problems that occur when samples are not randomly drawn from an identified population. Random sampling, however, is a meta-method assumption that appears to be routinely ignored or dismissed in research such as this.

Is the kind of causal statement that an RCT invites actually the statement that researchers who conduct RCTs of psychological treatments want to be able to make in all situations? Given the current design of psychological treatments, with different activities occurring in each session, it is difficult to believe that statements of causality are at the front of researchers’ minds during the conduct of this research. In a 12-session program of Cognitive Behavior Therapy (CBT), for example, what is it that can be identified as causal? Is it the sequence of activities, the number of sessions, the types of activities, or something else? It seems unlikely that such a non-specific approach would be as widespread as it currently is if more attention was paid to the causal statements that guide this research. What are the specific features and activity sequences in a manualized treatment protocol that are crucial to *cause* effective changes to occur? Does the client’s perspective on these aspects of the treatment protocol affect the assumptions of causality and the purported causal process?

A psychological treatment does not “work” independently of the clinician who is delivering the treatment and the client who is accessing it. That is, effectiveness is not an inherent property of the intervention (Carey, 2011; Carey et al., 2019). Effective outcomes are created by the client in interaction with the resources of the intervention. In the context of assigning causation to the treatment itself, “packages” of treatments have been developed that consist of pre-determined, but arbitrary, numbers of treatment sessions. Treatment manuals do not usually provide justification for the duration of treatments or why all the components of the package are necessary for effectiveness to be observed (Carey, 2011).

A specified number of sessions within a treatment protocol are very consistent with the “dose” model commonly applied in medicine (Carey, 2011). Understanding treatment as having a particular “dose” is another example of a meta-method assumption which has not been explicitly expressed and examined. Conceptualizing psychological treatments in terms of “doses,” however, and evaluating the potency of this dose with RCTs, has created two serious problems which will be discussed below. One arises from an error of interpretation and the other is a pragmatic problem.

Improvement and recovery for those people in non-treatment or control arms of RCTs is an example of how interventions cannot be claimed to cause effectiveness in the way that RCT methodologies assume. Invariably, in any RCT of psychological treatment, some participants in the control group will improve more than some participants in the treatment group. One review of wait list and cohort studies estimated that 53% of patients achieve remission from major depression within 12 months (Whiteford et al., 2013). Indeed, it has been estimated that only a third of individuals with mental health problems ever seek treatment (Alonso et al., 2004), so it might be assumed they recover without help. Taken together, these findings highlight how change is ongoing and dynamic, regardless of treatments that are offered. Many people seek informal help – from family or friends – for difficulties, and are likely to do so regardless of treatment (e.g., Brown et al., 2014).

Statements about mechanisms of change, in terms of the way in which they are stated – mostly statistically or conceptually – and the extent to which the stated mechanisms are linked to know biological structures and functions – are further meta-method assumptions that underpin many research designs but are seldom expressed explicitly. The regular scheduling of appointments of fixed duration, for example, is consistent with an assumption that change occurs in a linear, step-wise fashion. Despite evidence that change is frequently non-linear and unpredictable and can be “characterized by sudden disturbance and increased variability in system behavior before reorganization” (Hayes et al., 2007, p. 716), the regular scheduling of appointments remains a standard practice in routine clinical care.

Other work examining treatment as usual conditions of RCTs found that a third of patients achieved remission in this context (Kolovos et al., 2017). Treatment as usual was reported as rarely clearly defined and as a “dynamic treatment condition that is context dependent” (Kolovos et al., 2017, p. 78). This description highlights the difficulty of assigning causality to differences between conditions when the differences themselves have not been articulated. From a meta-method perspective a “dynamic treatment condition” does not provide sufficient detail to adequately inform a decision about appropriate methods to employ. Once again, attention to assumptions of causality at the design stage of the research process might help to address some of these problems.

### An Error of Interpretation

The first problem created through the assumption of psychological treatments providing different “doses” of assistance is an error of interpretation arising through flawed logic. By creating a treatment protocol of 16 sessions, and demonstrating, *via* RCTs, that 16 sessions of this particular treatment is associated with better outcomes than 16 sessions of something else (including nothing at all), people such as clinicians, health service managers, and policy makers, seem to have interpreted these results as indicating that 16 sessions are *necessary* for desirable results. Perhaps, if researchers were required, as part of making meta-methods explicit, to justify their decision to formulate a treatment protocol according to a particular number of sessions, these attitudes about how long treatment *should be* would not have arisen. Why are 16 (or some other number) “doses” required to *cause* reductions in psychological distress?

The necessity of 16 sessions, or any other pre-determined number of sessions, has never been established. Evidence that 16 sessions *can* be used to achieve good outcomes is, in no way, a demonstration that 16 sessions are *necessary* for good outcomes (Carey, 2011). This error, however, about what the results of an RCT actually mean, is currently reflected in policy documents such as the National Institute of Clinical Excellence (NICE) guidelines which state that “For all people with depression having individual CBT, the duration of treatment should typically be in the range of 16 to 20 sessions over three to four months” (National Institute for Health and Clinical Excellence, 2009, p. 28). In relation to the treatment of psychosis, NICE recommend that “CBT should be delivered on a one-to-one basis over at least 16 planned sessions” (National Institute for Health and Care Excellence, 2014, p. 22).

The recommendation of a specific number of sessions is especially surprising given that little agreement exists between treatment protocols as to what constitutes an “adequate dose” of CBT for psychosis (Addington and Lecomte, 2012). Guidelines such as these set unrealistic expectations that are not only costly, but lead to undue pressure in service delivery, as well as unacceptably long waiting times. There is also a risk that these guidelines have an unintended consequence of encouraging clinicians to focus more on retaining clients in treatment and less on helping clients achieve the outcomes that are important to them, irrespective of the number of sessions this takes.

### A Pragmatic Problem

The second problem created by the “dose” assumption is related to the first but is more of a pragmatic difficulty than an error of interpretation. The establishment of expectations about fixed durations of regularly scheduled treatment protocols creates an inflexibility in treatment delivery leading to inefficient services. There is a substantial and long-standing divide between the number of sessions that treatments are designed to be, and the number of sessions clients typically attend in routine clinical practice (Carey and Spratt, 2009). Furthermore, while treatment protocols are generally delivered in regularly scheduled sessions with weekly or twice-weekly intervals, clients in routine clinical practice rarely maintain a fixed attendance pattern (Carey and Spratt, 2009; Carey, 2011; Carey et al., 2013). This mismatch between the way in which treatments are offered and the way in which they are actually accessed lead to missed and cancelled appointments which, again, imposes an unnecessary financial burden on services and can compromise treatment. Therefore, there appears to be another important meta-method assumption which guides the design and research of psychological treatments but is actually discordant with routine clinical practice.

### Further Problems Created by the Preponderance of RCTs

While the problems mentioned above are serious enough, further problems created by the implicit assigning of causation to treatments inherent in an RCT methodology have resulted from the very narrow agenda established by RCTs.

RCTs are extremely valuable for establishing, under very specific conditions, if something achieves a result that something else does not. With regard to psychological treatments, however, many important questions remain unanswered and cannot be answered using RCTs. For example, we still do not understand how psychological treatments help in the ways that they do (Kazdin, 2009). That is, when someone accesses a psychological treatment and, during the course of that treatment, they experience an amelioration or alleviation of their psychological distress, we are still quite some way off from being able to coherently articulate the process by which the distress diminished. Perhaps, it is our unstated and erroneous meta-method assumptions that are guiding decisions about research designs which are impeding progress in this area.

Furthermore, evidence is lacking that identifies what elements of treatments are important for change (Cuijpers et al., 2019). Knowing why or how treatments work would enable us to both correct treatments efficiently when people have problems using them to create desired outcomes, as well as tailor treatments to meet the needs of individuals. It would also allow us to systematically design more helpful treatments. The mechanism of change is an example of a meta-method consideration because it applies whether someone is taking part in a pilot study interview, a clinical trial, or providing feedback in routine care. Whatever method is chosen, researchers should consider the relevance of mechanisms of change and should identify the mechanisms of change that they are implicitly subscribing to in the designs and methodologies they use.

In addition to not knowing how distress resolves, we still do not have a clear understanding of the reasons why it is that people access psychological treatment, though the barriers to accessing psychological help have been more clearly articulated (Salaheddin and Mason, 2016). Indeed, the latter line of research has developed a scale that only explores reasons for *not* seeking help, which is unlikely to shed light on reasons *for* seeking help. The assumption that people seek help for characteristic symptoms of diagnostic conditions may be another implicit, and erroneous, meta-method assumption. It is vital, however, to consider help seeking from the perspective of patients, as patients can be distressed by symptoms in idiosyncratic ways (Carey, 2017). Fried and Nesse (2015), for example, analyzed the symptom patterns of 3,703 people who had all been diagnosed with Major Depressive Disorder (MDD). They found 1,030 unique symptom profiles with 501 profiles being unique to 501 different individuals. Fried and Nesse (2015) concluded that the substantial individual variation in symptom patterns calls into question categorizing MDD as a discrete disorder and may explain some of the difficulty in demonstrating treatment efficacy when only sum-scores are considered.

What informs decision making about the timing of accessing psychological treatment is similarly unclear. Indeed, it is likely to be the case that there are a range of different reasons that people seek out psychological treatment but if we had dependable procedures for establishing what those reasons are, we could target our resources more precisely. Such procedures could form part of a program of research, or be used in the context of routine clinical practice. Again, this would lead to a more efficient use of resources.

### Operationalization Problems

Another problem created by the inappropriate assignment of causation to treatment occurs when specifying the Independent Variable (IV) and Dependent Variable (DV). As mentioned above, the underpinning, but rarely articulated, model of causation for RCTs is linear, and it is essential in the conduct of RCTs that IVs and DVs are clearly demarcated (Carey and Stiles, 2016). Unambiguously defining IVs and DVs would be another matter addressed by a meta-methods approach. With an RCT of psychological interventions, the IV is considered to be the treatment being tested, and the response of the client is taken to be the DV. When defined in this way, it can be readily appreciated that the IV and DV change during the course of treatment. Each session of treatment, for example, is different. A protocol of psychological treatment is unlike a protocol of pharmacological treatment in important ways. Moreover, the same session of a treatment protocol will be delivered differently by different clinicians or by the same clinician to different clients. Stiles and his colleagues use the term “responsive regulation” to refer to the phenomenon of the clinician and the patient adapting their conduct with each other in an ongoing way so that they each achieve their goals (Stiles et al., 2008). This entails a trial and error exploration so the client and therapist can identify what topics are pertinent to the client (Stiles et al., 1998). If “responsive regulation” was a meta-method assumption underpinning psychological treatment research, it is difficult to envisage how standardized treatment manuals would be used or fixed schedules of treatment delivery.

Apart from the practical difficulties mentioned above, it is not clear that IVs and DVs are the most appropriate way of conceptualizing variables gathered in studies of treatment effects with humans who have agency and purpose. Consequently, from this perspective, it is difficult to know the most appropriate outcomes to be measured when conducting an RCT. In RCTs of psychological interventions for psychosis, for example, reduction of psychotic symptoms is the most commonly measured primary outcome (Greenwood et al., 2010), despite the fact that there is evidence that symptoms of psychosis are only one of many possible sources of distress for this population and not necessarily the most important ones from the individual’s perspective (Griffiths et al., 2018). If an individual does not consider their symptoms to be distressing it would seem inappropriate to use symptom scores as the DV in research investigating treatment effectiveness. Issues such as these would be addressed by a meta-methods approach.

## An Alternative to the Current Trend – Introducing the Concept of Meta-Method

In order to make research more systematic, coherent, and scientific, it is essential that researchers begin to attend to important contextual considerations. Currently, our position is that these considerations guide research design and other research decisions invisibly but forcefully. By not recognizing and articulating coherent meta-method assumptions concerning causality, mechanisms, and so on, their influence on how research decisions are made cannot be evaluated. Researchers might develop more nuanced approaches to these matters if they were required to articulate and examine them explicitly.

Meta-method factors can be grouped into two broad categories with regard to the results of programs of research. The first category incorporates all matters relating to *what* outcomes a researcher is interested in obtaining. The second category includes those matters relating to *why* particular outcomes were obtained in the conduct of a particular study.

Specifically and clearly discerning the *what* and *why* factors in any proposed research will enable researchers to make important decisions about such matters as: which designs to use; how and when to mix methods; and the most appropriate measures to include. Perhaps the most important consideration will be the articulation of a coherent theoretical position that provides a strongly defensible rationale for the decisions that are made. The theory guiding the understanding of how therapy works can also be deployed to guide how it is tested. The section below describes one particular theory to illustrate the way in which a theoretical position can be employed in the manner just described.

### Perceptual Control Theory

Perceptual Control Theory (PCT; Powers, 2005) contends that humans, and indeed all living things, function according to the principle of negative feedback. When the results of different actions are fed back to the behaving agent, the agent can control environmental variables according to specified standards. While this control process has been recognized in areas such as homeostasis and cybernetics for many years (Wiener, 1948; Ashby, 1952), Powers’ unique insight was to realize that the same principle applied to *all* behavior, at all levels of complexity. Whether it is body temperature, the satisfaction of a relationship, or the progress of a career, that is being maintained, the same control process involving negative feedback loops applies (Carey et al., 2014). The feedback loops are negative feedback loops such that the task is always to *reduce* the difference between the standard that has been established and the current experience. Crucially, Powers argued that the standard is set by a process *internal* to the organism rather than inputted by a human controller as in the servo mechanisms described in cybernetics.

### Circular Causality

PCT provides a robust and integrative framework for research in both concept and method. The importance of feedback loops has already been highlighted but PCT also provides an alternative to linear conceptualizations of behavior. From a PCT perspective, circular causation, rather than linear causation, is a more appropriate model for understanding the activity of entities that live. Circular causation should not be confused with circularity. Its importance in understanding behavior has been recognized for over 100 years (Dewey, 1896) but, as a concept, it has been excruciatingly slow to gain traction in the life sciences. From the perspective of circular causality, CVs (controlled variables), rather than IVs and DVs, are more appropriate to investigate. CVs are those aspects of an individual’s perceived environment that do not vary according to environmental circumstances. Rather, the individual maintains these variables in a controlled state through acting on their environment.

The difference between DVs and CVs highlights a crucial departure by PCT from standard current approaches to researchers. Whereas it is standard practice to manipulate IVs to look for changes in the DVs, in PCT research, the focus is on identifying that variables (CVs) that *do not change* when IVs are manipulated. While standard research, therefore, focuses on the study of variability, PCT is the study of *invariance* which is more akin to research in the physical sciences. One reason for the power and precision of the physical sciences has been suggested to be the focus on invariance or the common, fundamental, underlying properties of seemingly distinct objects (Carey and Mansell, 2009, p. 128). Adopting this approach, therefore, might assist in improving the rigor and precision of research in the life sciences.

### Underlying Explanations Rather than Superficial Causes

An assumption underpinning the RCT methodology is that causality can be isolated by the manipulation of the treatment conditions compared to the control conditions. Thus, the same causality is extrapolated to future instances without gaining an understanding of what underlying process or processes brought about the effectiveness that was observed. The observations of beneficial effects of a treatment reflect what have been termed “consequences of underlying causes” (Powers, 2005). RCT researchers of psychological treatments, however, have been remarkably lax in the attention paid to underlying causes. Piantadosi (2005) asserts that “a clinical trial alone does not represent a scientific test of a therapy in the absence of a plausible mechanism of action for that therapy” (p. 19). Here Piantadosi (2005) is directly referring to meta-method considerations. Plausible mechanisms of relevance to psychological treatments, however, have been notoriously elusive, providing further evidence of the inadequacy of our current mono-methodological approach.

## Implications of Making Meta-Method Assumptions Based on PCT

The principles of PCT have important implications for the way in which research might be designed including the methods that will be used. In many ways, the activity of researchers who base their work on the principles of PCT will look similar to the activity of researchers who base their work on other theoretical principles. There will, however, be some important differences as well.

### Viewing Treatments as Resources Rather than Interventions

The notion that effectiveness is a consequence of patients interacting with treatments, positions the locus of change in a more appropriate position. As already noted, effectiveness is not something that is imposed on patients but something they create as they use the resources the treatment provides. This is a meta-method assumption that follows from PCT as outlined above, and the theory specifies that standards are internal - only the patient can define what resources are appropriate to enable them to move closer to the life they would like to live. If a resource is not being used by a patient, then that resource, for whatever reason, could be considered inappropriate and, therefore, not a resource at all, from that patient’s perspective. Considering treatments as resources, highlights how treatments do not *intervene* to force change in the way that a golf club projects a ball closer to its destination. This metaphor illustrates that the patients are unlike inanimate objects which can be directed from the outside. Instead, they are purposeful agents with their own sense of direction. If a clinician seeks to direct patients in a manner that is discrepant with the direction the patient has in mind, the likely reaction is *resistance to* the efforts of the clinician, resulting in what clinicians may interpret as drop out or treatment resistance. Psychological treatments, as resources, should enable patients to project themselves closer to whatever ultimate destination they intend.

### Ongoing Monitoring

The value of ongoing monitoring and evaluation is being increasingly recognized across different health fields (Carey et al., 2019). In the provision of psychological treatment, Routine Outcome Monitoring (ROM) has been demonstrated to be a useful innovation in assisting clients to maintain progress throughout treatment (Carey et al., 2019). In other fields, Continuous Quality Improvement (CQI) approaches have been adopted to improve service delivery (Carey et al., 2019).

While different names and methods are used in different contexts, the consistent theme with these approaches is the ongoing collection of data which is fed back to service providers so that treatments can be modified flexibly and responsively as required to maximize the likelihood of favorable outcomes (Carey et al., 2019). Clients are an important part of this process and data can be collected from a variety of sources in order to improve the accuracy of the information being accumulated. Research from this perspective is considered to be a means of learning rather than a way of making a judgment (Carey et al., 2019). The meta-method assumption following from PCT is that living things are constantly evaluating their experience against what standards they seek to achieve. PCT conceptualizes the process of living as an analogue, rather than as a digital (stop-start), process. For this reason, methods should construct research processes in an ongoing way rather than as occurring at arbitrary points chosen by the researcher.

### Perceptual Control Theory and Mixed Method Research

The underlying assumptions of PCT also offer a pertinent theoretical basis for conducting mixed method research. Proponents of mixed method approaches have extolled the advantages of mixing methods over using either quantitative or qualitative approaches alone (Johnson and Onwuegbuzie, 2004). Mixed methods are particularly appropriate when trying to develop complex interventions, such as psychological therapies (Craig et al., 2008; O’Cathain et al., 2013). Reconciling the apparently incompatible epistemologies that underpin qualitative and quantitative methodologies, however, the so-called “incommensurability thesis” (Symonds and Gorard, 2010), has presented challenges for researchers who wish to draw on the relative strengths of both approaches. From a PCT perspective, the meta-method assumption is that there is no inherent contradiction in adopting a position whereby an independent reality is assumed to exist, while simultaneously recognizing that this reality can only be known through our subjective perceptions. Using PCT to reconcile the theoretical positions of quantitative and qualitative approaches provides researchers with the opportunity to draw on a wider repertoire of research methodologies. Indeed, PCT can be considered a meta-theory which makes it especially suitable for assisting with meta-method assumptions. Designing programs of research that incorporate mixed methods might enable us to answer some of the previously intractable questions posed earlier in this article, such as *how* or *why* change occurs for people engaging with particular treatments.

The Method of Levels (MOL; Carey et al., 2009, 2013,  2017) is a cognitive therapy based on the principles of PCT that has used qualitative and quantitative approaches as well as relying on model building to test important theoretical propositions. Over a sustained period of time, MOL has been demonstrated to be an effective and efficient treatment which is flexible and responsive to clients’ varying needs. The development of this therapy, and a description of the different methods used, has been described elsewhere (Carey et al., 2017).

## Methodological Approaches That Emerge Directly from PCT

While PCT has a number of important implications for the meta-method assumptions that either implicitly or explicitly underpin all research, it also has some practical implications for methodological approaches.

### The Test for the Controlled Variable

A methodology called the Test for the Controlled Variable (TCV) has been proposed as a means of determining those aspects of the environment an individual is controlling, and has been fully described in a number of articles (Runkel, 1990; Marken, 2014). The process for the TCV involves establishing a hypothesis about a proposed variable an individual is controlling and then systematically applying disturbances or perturbations to the variable while observing the responses of the individual. If the individual responds by removing the effects of the disturbance and maintaining the variable in a particular state, this provides evidence of confirmation of the hypothesis. If, however, the individual does not remove the effects of the disturbance, then the hypothesis is disconfirmed and a new hypothesis is generated. The TCV is a systematic approach to the study of invariance alluded to above.

When considered from a PCT perspective, it can be appreciated that the controlled variable is both the cause, and a consequence, of a respondent’s actions, and their behavior is viewed as a manifestation of a closed loop system. This is a useful illustration of the concept of circular causality mentioned earlier. The TCV method emerges explicitly from PCT, rather than being based on looser assumptions about causality described earlier in relation to RCTs. As such, it helps to close the gap between a researcher’s meta-method assumptions and the experimental design being used. To date, the TCV has mostly been used in computer tracking tasks with extremely robust results (Marken, 2014). However, an interview technique is currently being developed to explore more abstract variables. This technique is described below.

### Model Building

The main approach to theory testing with PCT has been model building whereby functional models are constructed to simulate the phenomenon being investigated at the level of individual participants (Powers, 1989; Bourbon and Powers, 1993). The meta-method assumption being invoked here is that building functional models that simulate the behavior being investigated, is the most exacting form of model building. The term “model” is being used here in the same way that it would be used in engineering: “a precise quantitative proposal about the way some system operates in relation to its environment” (Bourbon and Powers, 1993, p. 51). By relying on simulations from models to test basic assumptions, PCT could be considered to be a theory that behaves. “A theory that behaves, that produces a stream of behavior, would seem in an intriguing way to fit better with Skinner’s chief criterion for a good theory than do many more common sorts of behavioral theory. Skinner has argued that a good behavioral theory is a theory on the same level as the behavior itself. What is closer to the level of a behavior stream of an organism than a behavior stream of a theory?” (Shimp, 1989, p. 170).

From a model building perspective, it is the degree of fit between the behavior being investigated and the behavior of the model that has been constructed to understand the investigated behavior, that is the outcome of interest rather, than a level of statistical confidence in the probability of making a Type I error. Simulations are already used extensively in the behavioral sciences (Fum et al., 2007) but this effort has not yet yielded the progress that has been expected or hoped for. This may be because the main tradition of modeling has been to simulate how *behavior is generated* rather than how *perceptions are controlled* to achieve and maintain inner standards (Mansell and Huddy, 2018). Here is another powerful illustration of the importance of meta-method assumptions. When the latter meta-method assumption is made, the level of fit between models and behavior is almost perfect.

One advantage of simulations is they can be used to predict population characteristics based on an underlying theory. This could include modeling whether individuals access therapy, and for how long, based on parameters in the model such as goals, or initial levels of distress. Model building, therefore, could be an additional methodology used to complement quantitative and qualitative approaches to further enhance the rigor of a program of research.

## Using a New Framework to Expand Our Research Efforts

A different conceptualization of treatment effectiveness, supported both by PCT and ongoing monitoring and evaluation, provides a valuable opportunity to more carefully consider the provision of psychological treatments for enhanced effectiveness and efficiency. In fact, it is our position, that enhancing the effectiveness and efficiency of psychological treatments will *only* be substantially improved by combining a range of different methodologies. By collecting both quantitative and qualitative data from different sources in an ongoing way, important questions about *what* changes occur during psychological treatment can be answered. Also, by incorporating model building methodologies with other methods, important questions about *why* and *how* these changes occur can be answered. Developing the TCV as a systematic approach to interviewing informed by PCT will provide an additional methodological resource for answering *what, why*, and *how* questions robustly.

## A TCV Interview

The use of the TCV as an approach to conduct interviews in qualitative research has the potential to improve our ability to understand the aspects of psychological treatments that people find most helpful. When combined with model building methodology, the TCV could also provide useful insights into the mechanisms of psychotherapeutic change, which are still poorly understood (Kazdin, 2009). Based on the same principles as in the example above, once an interviewer has formed a hypothesis about the variables that a participant might be controlling, it should be possible to ask questions that act as potential disturbances to that variable. Assuming that the interviewers’ hypothesis is correct, and the questions do act as a disturbance to a CV, the participant’s response is likely to serve the function of counteracting the effects of the question. If the participant is found to “push back” against the disturbance created by the question, the interviewer can have some confidence in their hypothesis. If no attempts to resist the disturbance are observed, the hypothesis is unlikely to be correct and another hypothesis should be formed.

Clearly, as with any approach to conducting interviews, it is envisaged that the TCV would be used sensitively. The aim is to generate a disturbance sufficient to test hypotheses regarding the variables that a participant might be controlling, not to cause the participant any discomfort or to withdraw from the conversation. As well as being ethically questionable, disturbances of excessive magnitude are likely to impede the overall interview process.

## Concluding Comments

Reorganizing our understanding of the locus of treatment effectiveness from inert treatment protocols to agentic individuals provides an opportunity to ask important questions about treatment effectiveness and efficiency. Combining different methodologies in the conduct of a program of ongoing monitoring and evaluation allows robust answers to these questions to be provided. An improvement in both the effectiveness and efficiency of psychological treatment, through explicit articulation and examination of important meta-method assumptions, will help to reduce the burden of psychological problems and will lead to more contented and productive individuals and communities.

## Author Contributions

TC developed the concept for the manuscript and drafted the initial version of the paper. VH and RG contributed information and technical expertise and edited, expanded, and modified the manuscript. TC provided additional information and final editing of the manuscript. All authors agree on the final version of the manuscript.

### Conflict of Interest Statement

The authors declare that the research was conducted in the absence of any commercial or financial relationships that could be construed as a potential conflict of interest.
